# The Prevalence of Irritable Bowel Syndrome after Severe Acute Respiratory Syndrome Coronavirus 2 Infection and Their Association: A Systematic Review and Meta-Analysis of Observational Studies

**DOI:** 10.3390/jcm12051865

**Published:** 2023-02-27

**Authors:** Ziyan Wang, Yinglong Peng, Minshan Chen, Liang Peng, Yongzhen Huang, Wei Lin

**Affiliations:** 1 Department of Gastroenterology, The First Affiliated Hospital of Guangzhou Medical University, Guangzhou 510120, China; 2 The First Clinical School, Guangzhou Medical University, Guangzhou 510120, China; 3 School of Medicine, South China University of Technology, Guangzhou 510006, China; 4 First Clinical Medical School, Southern Medical University, Guangzhou 510515, China; 5 School of Pediatrics, Guangzhou Medical University, Guangzhou 510182, China

**Keywords:** severe acute respiratory syndrome coronavirus 2, irritable bowel syndrome, coronavirus disease 2019, systematic review, meta-analysis

## Abstract

Aim: Investigate the prevalence of irritable bowel syndrome (IBS) after severe acute respiratory syndrome coronavirus 2 (SARS-CoV-2) infection and assess the association between IBS and SARS-CoV-2 infection. Methods: A systematic literature search for PubMed, Web of Science, Embase, Scopus, and the Cochrane Library was performed to identify all reports published before 31 December 2022. The confidence interval (CI), estimation effect (ES) of prevalence, and risk ratios (RR) were calculated to evaluate the prevalence of IBS after SARS-CoV-2 infection and their association. Individual results were pooled by the random-effects (RE) model. Subgroup analyses conducted a further investigation of the results. We employed funnel plots, Egger’s test, and Begg’s test to evaluate publication bias. Sensitivity analysis was performed for the assessment of the robustness of the result. Results: The data on IBS prevalence after SARS-CoV-2 infection were extracted from two cross-sectional studies and ten longitudinal studies from nineteen countries with 3950 individuals. The IBS prevalence after SARS-CoV-2 infection ranges from 3% to 91% in different countries, and the overall pooled prevalence of IBS following SARS-CoV-2 infection is 15% (ES: 0.15; 95% CI, 0.11–0.20; *p* = 0.000). The data on the association between IBS and SARS-CoV-2 infection were extracted from six cohort studies from fifteen countries with 3595 individuals. The risk of IBS increased following SARS-CoV-2 infection but was not significant (RR: 1.82; 95% CI, 0.90–3.69; *p* = 0.096). Conclusions: In conclusion, the overall pooled prevalence of IBS following SARS-CoV-2 infection was 15%, and SARS-CoV-2 infection increased the overall risk of IBS but was not statistically significant. Further extra high-quality epidemiological evidence and studies to clarify the underlying mechanism of IBS following SARS-CoV-2 infection are needed.

## 1. Introduction

Since the advent of coronavirus disease 2019 (COVID-19) in Wuhan in December 2019, this disease has rapidly spread worldwide and developed into a global pandemic. Researchers soon identified that severe acute respiratory syndrome coronavirus 2 (SARS-CoV-2), a positive-sense single-strand RNA virus with six open reading frames (ORFs)—such as replicase (ORF1a/ORF1b), spike, envelope, membrane, and nucleocapsid—is responsible for COVID-19 [1,2]. Angiotensin-converting enzyme 2 (ACE2) on the cell membrane is considered as the primary receptor that binds with spike protein of SARS-CoV-2; transmembrane protease, serine 2 (TMPRSS2) can promote fusion of the cell membrane and SARS-CoV-2-ACE2 complex to assist SARS-CoV-2 entry into the cell [3,4]. It has been well researched that SARS-CoV-2 causes substantial respiratory pathological damage and results in COVID-19 with typical manifestations such as fever, cough, pneumonia, and acute respiratory distress syndrome [1,5,6]. However, researchers have found many extrapulmonary manifestations, ranging from the gastrointestinal and hepatic system, immunological system, hematologic system, and cardiovascular system to the renal system [6]. It has been reported that the occurrence of gastrointestinal symptoms was significantly higher in COVID-19 patients (59.7%) than in participants without COVID-19 (43.2%) (*p* < 0.001) based on the results from a prospective, multicenter, controlled study carried out by Marasco and their colleagues [7]. A meta-analysis investigated digestive involvement in COVID-19 patients and reported that symptoms such as nausea, vomiting, diarrhea, and loss of appetite exist in COVID-19 patients [8]. These findings reveal that SARS-CoV-2 may be associated with gastrointestinal disorders.

IBS, as defined by the Rome IV criteria, is characterized by recurrent abdominal pain occurring at least once a week on average for the past three months, along with two or more associated symptoms, such as those related to defecation, changes in stool frequency, or changes in stool appearance, with the symptom onset occurring at least six months before diagnosis and persisting for the past three months [9,10]. Conversely, the Rome III criteria define IBS as recurring abdominal pain that has transpired at least three times per month over a period of three months [9,11]. Researchers have estimated that IBS affects one in ten people around us and substantially burdens individual costs and quality of life [12]. For instance, in China, the healthcare budget for the entire nation allocates 3.3% towards IBS [13]. According to a survey conducted by Ballou et al., patients diagnosed with IBS frequently avoid locations that do not have easily accessible bathrooms, face challenges when making plans, and feel hesitant to leave their homes or travel [14]. Additionally, they tend to refrain from sexual activity, have difficulty focusing, and experience self-consciousness [14]. Infection is one of the most important pathogeneses that causes IBS [15,16,17,18]. It has been reported that bacterial, viral, and protozoal factors result in post-infection irritable bowel syndrome (PI-IBS) [18]. Some observational studies have specifically described or investigated the onset of PI-IBS following COVID-19 but failed to achieve universally accepted results [19,20,21,22,23,24]. Moreover, some researchers reviewed the current studies and summarized the potential mechanisms of IBS after SARS-CoV-2 infection [2,25]. In fact, there is a lack of information about the prevalence of IBS following SARS-CoV-2 infection and their association. Thus, it is necessary to investigate the prevalence of IBS following SARS-CoV-2 infection and their association by systematically reviewing the current studies.

## 2. Methods

We implemented this study according to the guidelines of Meta-analysis of Observational Studies in Epidemiology (MOOSE) and Preferred Reporting Items for Systematic Reviews and Meta-Analyses (PRISMA) [26,27]. This study is registered in the International Platform of Registered Systematic Review and Meta-analysis Protocols (registration number INPLASY2022110138).

### 2.1. Eligibility Criteria

There are two phases of the selection process. Phase 1 involves the selection of the studies that reported the number of IBS cases following infection of SARS-CoV-2, for which the IBS prevalence post-SARS-CoV-2 infection is calculated. Phase 2, based on the included studies in phase 1, involves the selection of the studies that reported the number of IBS cases in the control group with no SARS-CoV-2 infection, for which the relationship between IBS and SARS-CoV-2 infection is investigated. Inclusion criteria: (a) population: participants from community or hospital; (b) exposure: SARS-CoV-2 infection history; (c) primary outcome: the prevalence of IBS after SARS-CoV-2 infection; (d) study design: an observational study. Phase 1: exclusion criteria: (a) studies irrelevant to humans; (b) article type: abstract, review, editorial, comment, reply, and note; (c) the study did not investigate IBS symptoms after SARS-CoV-2 infection. Phase 2: exclusion criteria: (a) studies without a control group: participants with no SARS-CoV-2 infection; (b) studies that could not calculate the secondary outcome: the risk ratio (RR) between IBS and SARS-CoV-2 infection.

### 2.2. Study Selection

Two investigators (Z.W. and Y.P.) designed the search strategy together and obtained the agreement of all researchers for the search strategy. These two investigators (Z.W. and Y.P.) independently searched databases such as PubMed, Web of Science, Embase, Scopus, and the Cochrane Library for records until 31 December 2022. No language restrictions were used. We applied the following text and the MeSH terms: “SARS-CoV-2”, “COVID-19”, and “irritable bowel syndrome.” The specific search strategy used in these databases is presented in Supplementary Materials S1. Two investigators (Y.P. and Z.W.) separately implemented the search. We imported these records into Endnote X20 (Clarivate Analytics, Philadelphia, PA, USA). The automation tool Endnote X20 deleted duplicate records, and Z.W. double-checked the records manually. Two investigators (Z.W. and Y.P.) evaluated whether these records met the eligibility criteria by reading their full texts and resolved the differences through a discussion with L.P.

### 2.3. Data Extraction and Quality Assessment

We abstracted the following data from each included study: (a) author; (b) year; (c) follow-up period; (d) region; (e) study design; (f) IBS diagnostic criteria; (g) SARS-CoV-2 detection method; (h) age information; (i) gender information; (j) the number of IBS patients after the infection of SARS-CoV-2; and (k) the number of IBS patients in the control group. The Newcastle–Ottawa scale (NOS) and the quality assessment scale set by the Agency for Healthcare Research and Quality (AHRQ, Rockville, MD, USA) are two of the most widely used scales to evaluate observational studies (NOS for longitudinal study and case–control study; AHRQ for cross-sectional study). We defined a high-quality study as attaining over 70% on the respective rating scale, which means seven or more out of nine scores for the NOS and eight or more out of eleven scores for the AHRQ scale. M.C. and Y.P. carried out the quality assessment irrespectively and resolved differences via discussion with Z.W.

### 2.4. Data Synthesis and Statistical Analysis

The IBS prevalence after SARS-CoV-2 infection was calculated and is presented as effect size (ES). We calculated the total RR of all cohort studies and the RR of each cohort study. Moreover, the 95% confidence intervals (CI) and *p*-value of all included studies were calculated. The I^2^ and *p* values were employed to present the effect of the heterogeneity test. When the heterogeneity test’s I^2^ > 50% or *p* < 0.1, we considered the heterogeneity to be significant [28,29,30]. In this study, we used the random-effects (RE) model for calculations. We employed subgroup analyses to further investigate the study results from these three aspects: (a) region, (b) study design, or (c) quality of the study. We considered this aspect as a possible heterogeneity source when the pooled I^2^ of each group of the subgroup analysis was less than the overall pooled I^2^ of the subgroup analysis. The sensitivity analysis evaluated the robustness of the results via consecutive omission of each included study. Publication bias was evaluated by funnel plot, Egger’s test, and Begg’s test [31,32]. All *p* values are two-sided. Moreover, *p* < 0.05 was taken as statistically significant, except for the *p*-value of the heterogeneity test. Stata 15 (Stata Corp, College Station, TX, USA) was employed for statistical analysis.

## 3. Results

### 3.1. Study Selection

**Figure 1:**
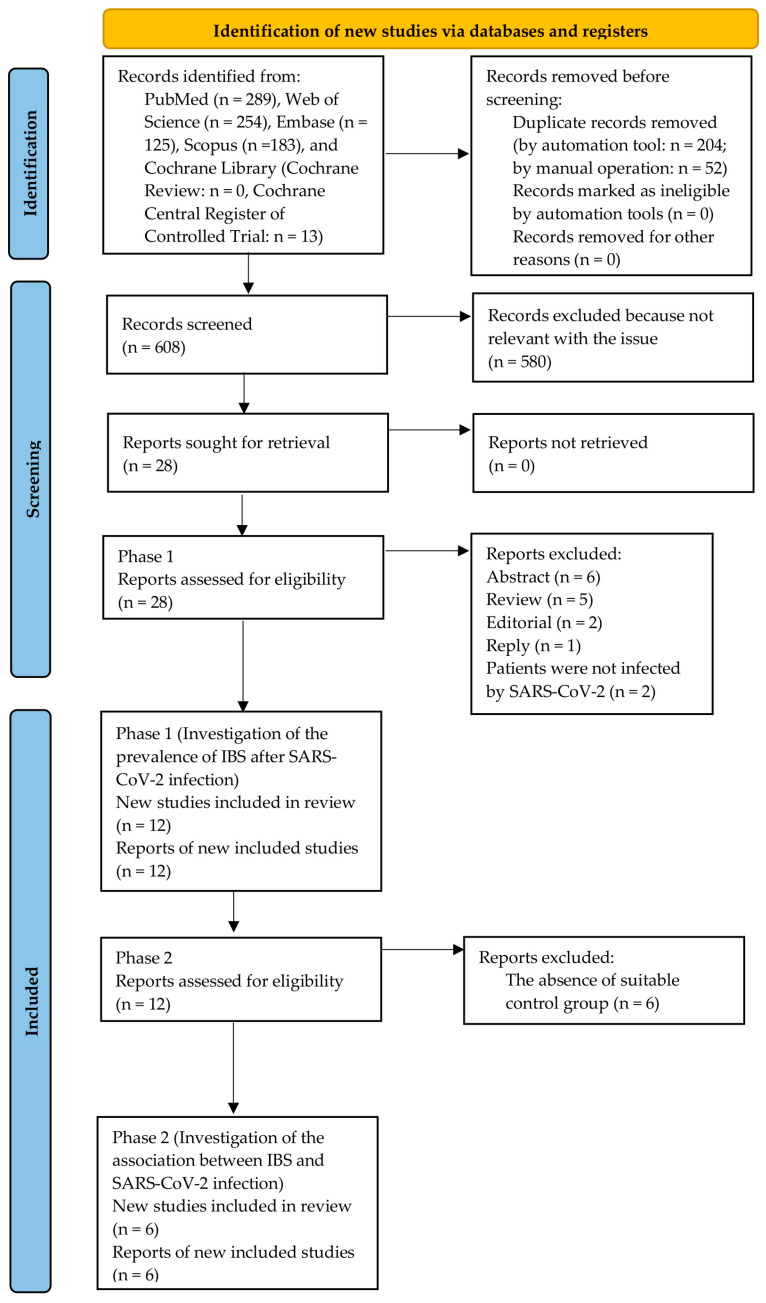
PRISMA study selection flow [26].

We selected 864 records from PubMed (n = 289), Web of Science (n = 254), Embase (n = 125), Scopus (n = 183), and the Cochrane Library (n = 13). Next, two hundred and fifty-six duplicate records were deleted. A total of 580 records were removed because they did not become relevant to this issue. We then read the full articles of the rest of the 28 records to confirm whether these records met the eligibility criteria in phase 1. We excluded sixteen studies due to the reasons like abstracts, reviews, editorials, replies, or involving patients without SARS-CoV-2 infection. In phase 1, twelve studies were included in the meta-analysis for investigating the IBS prevalence after SARS-CoV-2 infection [19,20,21,22,23,24,33,34,35,36,37,38]. Six studies were excluded in phase 2 because of the absence of a suitable control group. In phase 2, six studies were included in the meta-analysis for investigating the association between IBS and SARS-CoV-2 infection [22,23,34,35,36,38]. The PRISMA study selection flow is given in Figure 1.

### 3.2. Study Characteristics

Two cross-sectional studies with 397 individuals and ten longitudinal studies with 5639 individuals were included. Six of the longitudinal studies followed up for six months, one for three to six months, one for five months, one for twelve months, and one had a follow-up period of six to twelve months. Ten of the included studies used the Rome IV criteria for IBS diagnosis, while one employed the Rome III criteria, and one utilized a self-designed questionnaire. Out of the twelve studies analyzed, only seven utilized nucleic acid amplification tests (NAATs) such as the polymerase chain reaction (PCR), reverse transcriptase-polymerase chain reaction (RT-PCR), and cartridge-based nucleic acid amplification test (CBNAAT) to confirm SARS-CoV-2 infection, while three studies employed a variety of SARS-CoV-2 detection methods. The remaining two studies did not report the specific testing method used to confirm SARS-CoV-2 infection. Participants in eleven included studies were adults, while one study included all children. The detailed characteristics of all included studies are displayed in Table 1. The quality assessment scores comprising all included studies appear in Supplementary Materials S2.

### 3.3. Prevalence of IBS Following SARS-CoV-2 Infection

**Figure 2:**
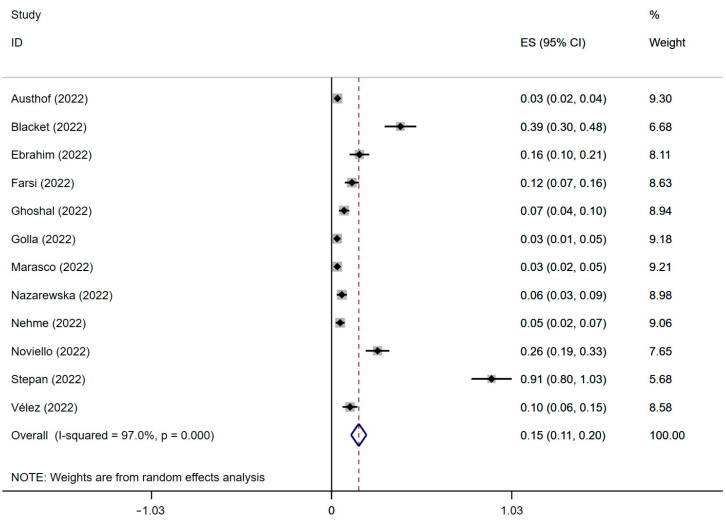
Forest plot of the IBS prevalence after SARS-CoV-2 infection [19,20,21,22,23,24,33,34,35,36,37,38]. IBS, irritable bowel syndrome; SARS-CoV-2, severe acute respiratory syndrome coronavirus 2; ES, effect size.

The results were based on the data of twelve studies with 3950 individuals from nineteen countries. The prevalence of IBS following SARS-CoV-2 infection is presented in Figure 2. The result of the significance test is displayed in Supplementary Materials S3. The summary prevalence of IBS following SARS-CoV-2 infection was 15% (ES: 0.15; 95% CI, 0.11–0.20; *p* = 0.000; heterogeneity: *p* = 0.000; I^2^ = 97.0%), with the prevalence of each group falling within the range from 3% to 91%.

### 3.4. Prevalence of IBS following SARS-CoV-2 Infection Classified by Region and Study Design

**Figure 3:**
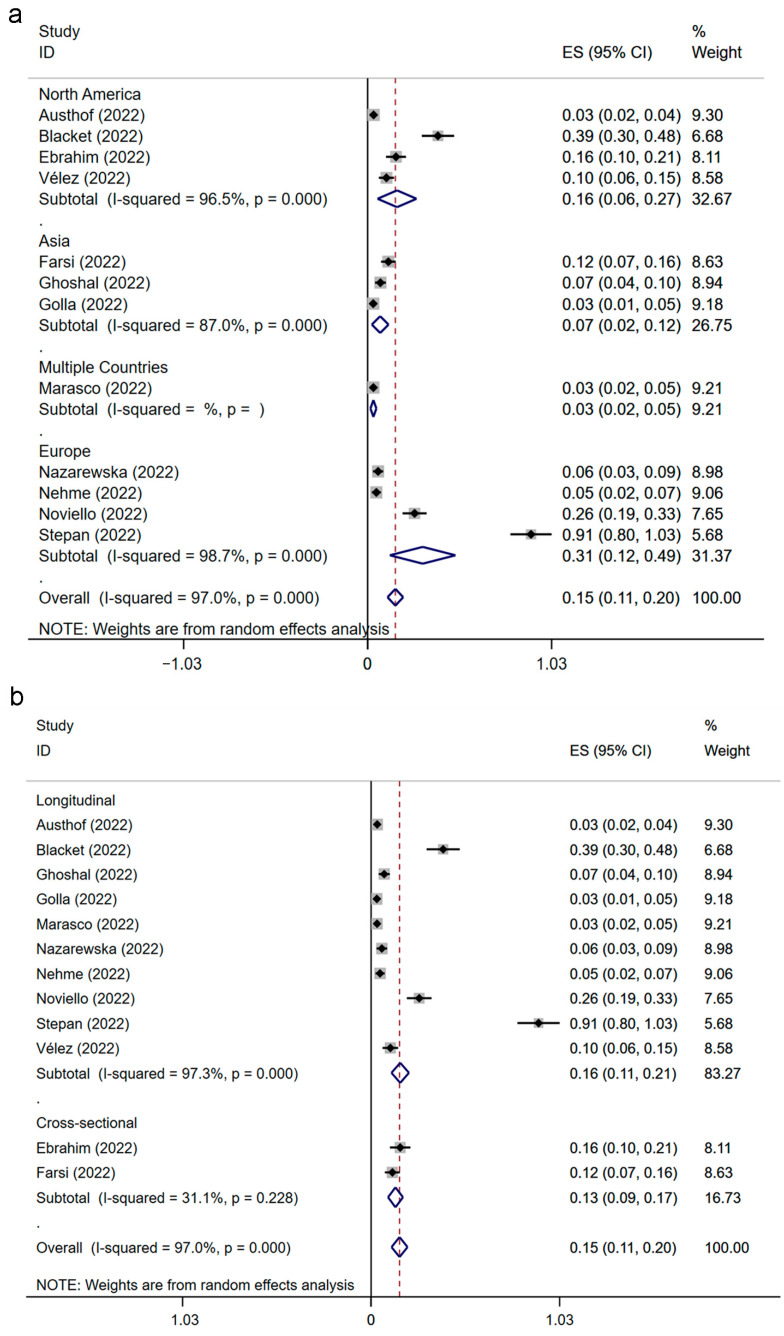
Subgroup analysis of IBS prevalence after SARS-CoV-2 infection [19,20,21,22,23,24,33,34,35,36,37,38]. Forest plots classified by region (**a**) and study design (**b**). IBS, irritable bowel syndrome; SARS-CoV-2, severe acute respiratory syndrome coronavirus 2; ES, effect size.

The prevalence estimations of IBS after SARS-CoV-2 infection were researched by us from the region and study design (in Figure 3). The significance test is shown in Supplementary Materials S3. The prevalence estimation of post-SARS-CoV-2-infection IBS was 31% in Europe (ES: 0.31; 95% CI, 0.12–0.49; *p* = 0.001; heterogeneity: *p* = 0.000; I^2^ = 98.7%), which was much higher than that in North America (ES: 0.16; 95% CI, 0.06–0.27; *p* = 0.003; heterogeneity: *p* = 0.000; I^2^ = 96.5%) and in Asia (ES: 0.07; 95% CI, 0.02–0.12; *p* = 0.003; heterogeneity: *p* = 0.000; I^2^ = 87.0%). In addition, a multinational study reported the prevalence of IBS after COVID-19 was 3% (ES: 0.03; 95% CI, 0.02–0.05; *p* = 0.081) [38]. The prevalence of IBS after SARS-CoV-2 infection in cross-sectional studies was 13% (ES: 0.13; 95% CI, 0.09–0.17; *p* = 0.000; heterogeneity: *p* = 0.228; I^2^ = 31.1%). In the longitudinal-design group, the prevalence of IBS after SARS-CoV-2 infection was 16% (ES: 0.16; 95% CI, 0.11–0.21; *p* = 0.000; heterogeneity: *p* = 0.000; I^2^ = 97.3%).

### 3.5. The Association between IBS and SARS-CoV-2 Infection

**Figure 4:**
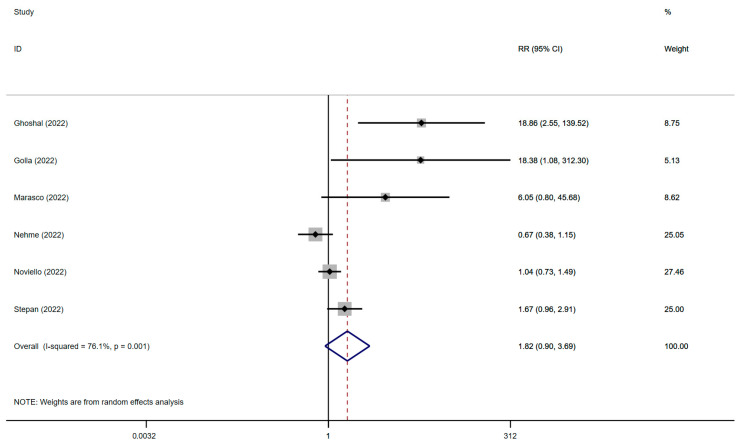
Forest plot of the association between IBS and SARS-CoV-2 infection [22,23,34,35,36,38]. IBS, irritable bowel syndrome; SARS-CoV-2, severe acute respiratory syndrome coronavirus 2; RR, risk ratio.

The results relied on data from six studies from fifteen countries with 3595 individuals. The relationship between IBS and SARS-CoV-2 infection is displayed in Figure 4, and the significance test is presented in Supplementary Materials S3. Our observation suggested that SARS-CoV-2 infection was potentially associated with IBS (RR: 1.82; 95% CI, 0.90–3.69; *p* = 0.096; heterogeneity: *p* = 0.001; I^2^ = 76.1%).

### 3.6. The Association between IBS and SARS-CoV-2 Infection Classified by Region and Study Quality

**Figure 5:**
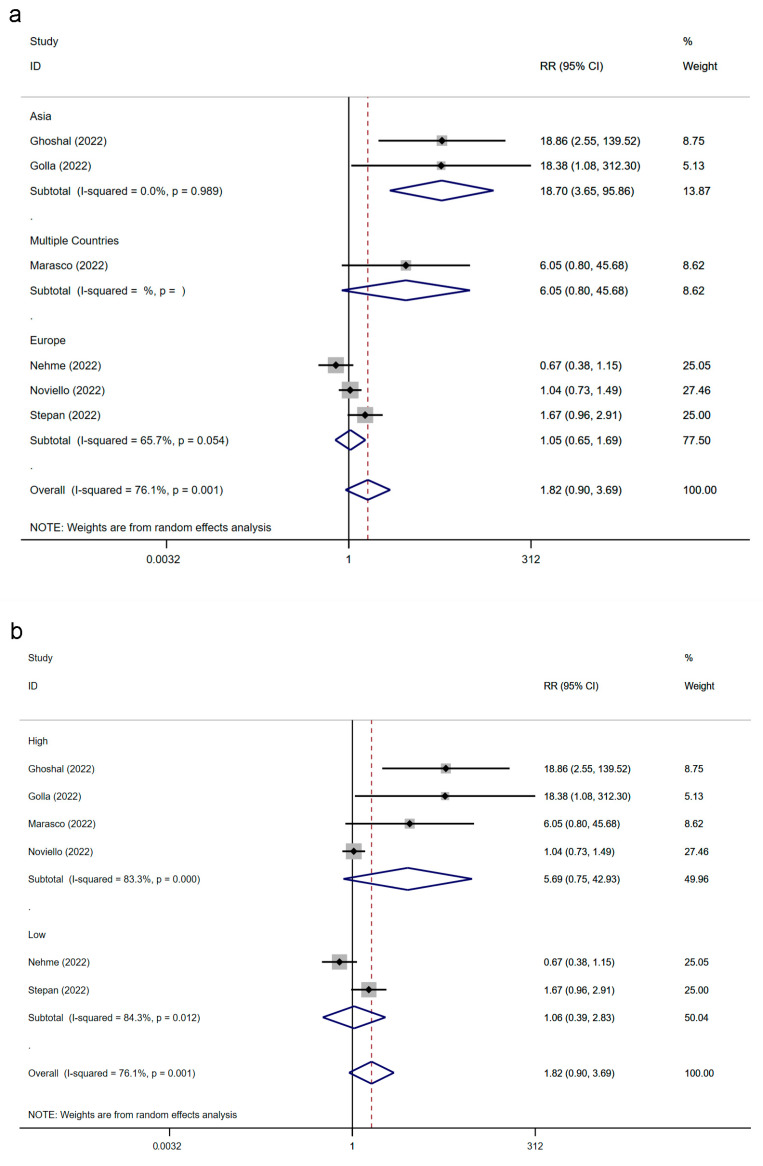
Subgroup analysis of the association between IBS and SARS-CoV-2 infection [22,23,34,35,36,38]. Forest plots classified by region (**a**) and study quality (**b**). IBS, irritable bowel syndrome; SARS-CoV-2, severe acute respiratory syndrome coronavirus 2; RR, risk ratio.

We analyzed the relationship between IBS and SARS-CoV-2 infection from the region and the quality of the included studies, as shown in Figure 5 and Supplementary Materials S3. In Asia, the two studies reported that the pooled RR of IBS following SARS-CoV-2 infection was 18.70; the result was statistically significant, and the level of heterogeneity was low (95% CI, 3.65–95.86; *p* = 0.000; heterogeneity: *p* = 0.989; I^2^ = 0.0%). However, the other three European studies reported that SARS-CoV-2 infection did not significantly increase the risk of IBS, and their results had high heterogeneity (RR: 1.05; 95% CI, 0.65–1.69; *p* = 0.841; heterogeneity: *p* = 0.054; I^2^ = 65.7%). Additionally, the RR of the multinational study carried out by Marasco and colleagues was 6.05 (95% CI, 0.80–45.68; *p* = 0.081). Notably, the region classification decreased the overall heterogeneity (*p* = 0.001; I^2^ = 76.1%). We found that the RR of high-quality studies (RR: 5.69; 95% CI, 0.75–42.93; *p* = 0.092; heterogeneity: *p* = 0.000; I^2^ = 83.3%) was higher than that of low-quality studies (RR: 1.06; 95% CI, 0.39–2.83; *p* = 0.915; heterogeneity: *p* = 0.012; I2 = 84.3%), but neither was statistically significant.

### 3.7. Publication Bias Assessment

**Figure 6:**
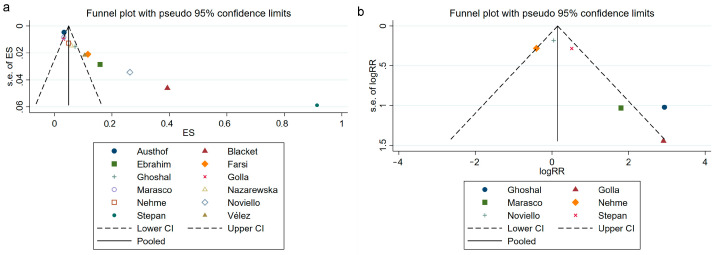
Publication bias evaluation. Funnel plot of the IBS prevalence after SARS-CoV-2 infection (**a**) [19,20,21,22,23,24,33,34,35,36,37,38]. Funnel plot of the association between IBS and SARS-CoV-2 infection (**b**) [22,23,34,35,36,38]. IBS, irritable bowel syndrome; SARS-CoV-2, severe acute respiratory syndrome coronavirus 2; ES, effect size; RR, risk ratio.

The funnel plots are shown in Figure 6. The plots of Egger’s test and Begg’s test are displayed in Supplementary Materials S4. We confirmed significant asymmetry in the analysis reporting the prevalence of IBS following SARS-CoV-2 infection. We then further confirmed this asymmetry with Begg’s test (*p* = 0.000) and Egger’s test (*p* = 0.001). We found no publication bias in the analysis of the relationship between IBS and SARS-CoV-2 infection by the symmetry of the funnel plots. Further to this, the findings of Egger’s test (*p* = 0.133) and Begg’s test (*p* = 0.054) both supported this finding.

### 3.8. Sensitivity Analysis

**Figure 7:**
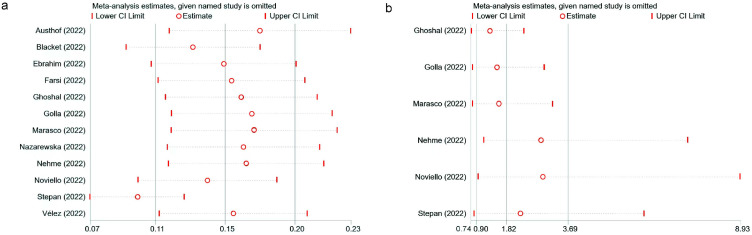
Sensitivity analysis. The IBS prevalence after SARS-CoV-2 infection (**a**) [19,20,21,22,23,24,33,34,35,36,37,38] and the association between IBS and SARS-CoV-2 infection (**b**) [22,23,34,35,36,38].

Visual inspection can identify that the result of the analysis of the prevalence of IBS following SARS-CoV-2 infection had poor stability (Figure 7a); the study of Stepan et al. affected the pooled prevalence significantly [36]. The sensitivity analysis result (Figure 7b) supported the stable association between IBS and SARS-CoV-2 infection.

## 4. Discussion

This meta-analysis comprised 12 studies and investigated a large-scale population with 6036 participants from 19 regions to inspect the prevalence of IBS following SARS-CoV-2 infection and their association. The overall pooled prevalence of IBS following infection of SARS-CoV-2 was 15%, which was supported by the result of a previously published study [39]. In general, the global prevalence of IBS is between 3.8% and 9.2%, according to the criteria of Rome III or Rome IV [40]. Therefore, SARS-CoV-2 infection increases the prevalence of IBS compared with the global average IBS prevalence. However, it has been reported that Rome IV IBS is not as stable as Rome III IBS [41]. Researchers may underestimate the prevalence of IBS after SARS-CoV-2 infection if they use the Rome IV criteria instead of the Rome III criteria [9,40]. This means that our estimated prevalence of IBS after COVID-19 was perhaps more conservative than the actual prevalence because most of the included studies employed the Rome IV criteria, which is also supported by the results from Nazarewska et al. [24]. Klem et al. found that the pooled prevalence of PI-IBS after infectious enteritis is 10.1% at 12 months and 14.5% at more than 12 months [18]. More specifically, the PI-IBS prevalence of bacterial enteritis, parasitic enteritis, and viral enteritis was 13.8%, 41.9%, and 6.4%, respectively [18]. This evidence indicates that COVID-19 may pose a higher risk in causing IBS than infectious enteritis. Given the global COVID-19 pandemic, this situation could substantially burden the preliminary healthcare system, society, and economy. In this study, the prevalence of IBS following SARS-CoV-2 infection varied from 3% to 91% among different countries. According to the results of subgroup analysis classified by region, we found the prevalence of IBS following SARS-CoV-2 infection to be 31% in Europe, 16% in North America, and 7% in Asia. These findings indicate a regional difference in the prevalence of post-SARS-CoV-2-infection IBS, which is also supported by the observed regional differences in the global burden of IBS from the previously published investigation [40,42]. Moreover, we observed a higher pooled prevalence of IBS after SARS-CoV-2 infection in the longitudinal studies than in the cross-sectional studies, and the heterogeneity of cross-sectional studies is less than that of longitudinal studies. The cross-sectional study intercepts the prevalence of post-SARS-CoV-2-infection IBS at a particular time point and is less affected by follow-up time, which has less heterogeneity of the results but fails to observe all IBS events after infection. In contrast, the longitudinal study is sensitive to the follow-up time; thus, the results vary widely, which causes high heterogeneity of the results while it approaches the final number of IBS events after SARS-CoV-2 infection. Schmulson and colleagues suggested that the diagnosis of post-COVID-19 IBS should be established 6 months after the onset and fulfill the symptoms of Rome IV IBS for the last 3 months [43]. Therefore, researchers should choose a suitable follow-up time (maybe more than 6 months) to investigate the prevalence of IBS after SARS-CoV-2 infection to achieve final results with higher accuracy and less heterogeneity.

Although we found that SARS-CoV-2 infection increases the prevalence of IBS, the relationship between IBS and SARS-CoV-2 infection remains unclear. In general, we found that SARS-CoV-2 infection increases the risk of IBS, but we observed a nonsignificant association between IBS and infection of SARS-CoV-2. Interestingly, we found that IBS is significantly associated with SARS-CoV-2 infection in Asia but not in Europe. Recent studies found that the outcome after SARS-CoV-2 infection is associated with race, lifestyle, socioeconomic status, and vaccination status [44,45,46]. Thus, we suspected that this regional difference may be associated with the difference in these multiple factors between Europe and Asia. Moreover, the heterogeneity of Asian and European studies was less than the pooled heterogeneity of the meta-analysis, which suggested that regional differences may be a major source of heterogeneity. In the subgroup classified by study quality, the heterogeneity of the low-quality and high-quality studies was higher than the overall pooled heterogeneity. We considered the limited number of included studies and regional differences may cause this high heterogeneity. The clinical manifestation and comorbidities of COVID-19 were associated with its severity [47,48]. Chen and colleagues reported that COVID-19 inpatients had a higher prevalence of long COVID syndrome compared with COVID-19 outpatients [49]. Consequently, it is possible that the severity of COVID-19 may affect the association between IBS and SARS-CoV-2 infection.

Previous reviews summarized that dysbiosis of the gut microenvironment, dysfunction of the gut–brain axis, visceral hyperalgesia caused by post-inflammatory/post-infectious neuroplastic changes, altered gut motility, and genetics are the central mechanisms of IBS [15,17]. The gut infection has been accepted as a pathogenesis that induces the onset of IBS [15,16,17,18]. Interestingly, SARS-CoV-2 infection may be associated with some mechanisms of IBS. In early 2020, Xiao et al. demonstrated gastrointestinal infection of SARS-CoV-2 and reported high expression of ACE2 protein in glandular cells of epithelia from the stomach, duodenum, and rectum [50]. Moreover, Ziegler et al. reported the co-expression of ACE2 and TMPRSS2 in ileal absorptive enterocytes [51]. These results reveal the underlying molecular mechanism by which SARS-CoV-2 infects gastrointestinal cells. Furthermore, they observed infiltrating plasma cells and lymphocytes of interstitial edema in the gastric, duodenum, and rectal lamina propria in COVID-19 patients, demonstrating that COVID-19 results in damage to the gut barrier by inducing gastrointestinal inflammation [50]. This finding suggests a potential increase in gut permeability. Moreover, in many common gastrointestinal disorders, especially IBS, gastrointestinal inflammation has been reported to be associated with the alteration of gut neuroplasticity [52]. Sinhamahapatra et al. demonstrated that rectal stimulation triggers visceral hyperalgesia, which can be recorded by the cerebral evoked potential [53]. Therefore, we speculated that SARS-CoV-2 infection causes visceral hyperalgesia after post-inflammation neuroplastic changes in the gastrointestinal tract, particularly the rectum. Recently, Liu et al. reported that COVID-19 affects the gut microbiome composition, resulting in increased levels of Ruminococcus gnavus, Bacteroides vulgatus and decreased levels of Faecalibacterium prausnitzii [54]. The alteration of gut microbiome composition is significantly associated with persistent syndrome for even six months after the clearance of SARS-CoV-2, whereas the syndrome includes neurological symptoms, hair loss, fatigue, respiratory symptoms, gastrointestinal symptoms, and musculoskeletal symptoms [54]. Notably, certain treatments for COVID-19 patients, such as broad-spectrum antibiotics, corticosteroids, and antiviral drugs, may have an impact on the composition of the gut microbiome, which is considered to be a potential factor in the onset of IBS [2,55,56,57]. As a result, in general, COVID-19 causes increased permeability of the gut barrier, and dysbiosis of the gut microbiome reflects dysfunction of the gut microenvironment. Moreover, SARS-CoV-2 infection and the restriction measures of the COVID-19 pandemic have increased the incidence of psychological, psychiatric, and neuropsychiatric syndromes from delirium, anxiety, depression, and post-traumatic stress disorders to encephalitis [38,58,59,60,61]. The evidence provided by Toubasi et al. indicated that COVID-19 patients who had mental disorders had increasing incidences of mechanical ventilation, ICU admission, and mortality, suggesting that psychological and psychiatric factors affect the development and outcome of COVID-19 [62]. These pieces of evidence show the potential role of the gut–brain axis in the interaction between COVID-19 and mental health. We surmised that many factors, such as the severity of COVID-19 and medical requirements, influence the perception and intensity of COVID-19 via the gut–brain axis, which may affect the association between IBS and SARS-CoV-2 infection. COVID-19 may predispose individuals to IBS by perturbing the gut–brain axis, which plays a crucial role in the progression of IBS [17]. Taken together, although many unsolved problems exist, we speculated that visceral hyperalgesia after post-inflammation neuroplastic changes, dysfunction of the gut microenvironment, and perturbance of the gut–brain axis by mental disorders may participate in the onset or development of IBS after SARS-CoV-2 infection.

This research has some strengths. First, this is the first systematic review and meta-analysis specifically to examine the prevalence of IBS post-SARS-CoV-2 infection and their association, providing qualitative and quantitative evidence of IBS following SARS-CoV-2 infection. Second, this study is a large-scale investigation with 6036 individuals. Third, we highlight the variations in prevalence and the strength of this association classified by region, study design, or study quality. Fourth, we investigated five widely used databases to improve the representativeness of the included literature.

This research has some noteworthy limitations. First, the significant heterogeneity observed in this study may potentially impact the reliability of the prevalence of IBS following SARS-CoV-2 infection and its association with the virus. Although we did not fully elucidate the source of heterogeneity, fortunately, we identified that regional differences may be the primary source of heterogeneity in the association between IBS and SARS-CoV-2 infection. Second, some demographic data from the included studies were lost, which may affect the accuracy of the results. Third, some included studies were single-center or had a limited study population, which may result in a lack of representation of the conclusion and allow extreme values to affect the results of the study. Fourth, we did not consider the influence of sex, age, diagnostic criteria, testing method, the severity of COVID-19, medical requirements, and psychological factors, which may cause confounding bias. Fifth, COVID-19 infected patients with different variants of SARS-CoV-2 [63]. Despite this, we did not analyze how the variant differences of SARS-CoV-2 have affected the study results. Sixth, due to the limited follow-up time of the included studies, this study only included IBS events within 12 months after SARS-CoV-2 infection.

## 5. Conclusions

In conclusion, we investigated the available studies and revealed that the overall pooled prevalence of IBS following SARS-CoV-2 infection was 15%, and SARS-CoV-2 infection increased the overall risk of IBS but was not statistically significant. This study indicates that IBS is ubiquitous after SARS-CoV-2 infection, but we cannot confirm that SARS-CoV-2 infection is a risk factor for IBS. Although there remain many unanswered questions surrounding IBS following SARS-CoV-2 infection, our study first comprehensively summarizes the current evidence and investigates the IBS prevalence following SARS-CoV-2 infection and the magnitude of their association. Further studies, such as prospective cohort studies and basic medical studies, are needed to provide additional high-quality epidemiological evidence or clarify the underlying mechanism of post-SARS-CoV-2-infection IBS.

## Figures and Tables

**Table 1:** The characteristics of the included studies.

Author	Year	Follow-Up Period	Region	Study Design	IBS Diagnostic Criteria	SARS-CoV-2 Detection Method	Age Information	Gender	The Number of IBS Patients Following SARS-CoV-2 Infection	The Number of IBS Patients in Control Group (Without SARS-CoV-2 Infection)	Study Quality
Austhof [19]	2022	6 months	USA	Longitudinal	Rome IV	PCR test	Mean age (SD): No GI symptoms: 45.0 (15.9) years; GI symptoms: 42.7 (15.6) years	Male:487 Female:978 Non-Binary:7 Transgender female: 2 Transgender male: 1	49 of 1475	NR	Low
Blackett [20]	2022	6 months	USA	Longitudinal	Rome IV	PCR test/Antibody test/Symptom/Unclear methods	>18 years old	Male:28 Female:84	44 of 112	NR	High
Ebrahim [33]	2022	NA	USA and Canada	Cross-sectional	Rome IV	PCR test or antibodies test	Age- group distribution was ≤65 years old: 79%, >65 years old: 4%, did not report their age: 17%	Male:14% Female:70% 16% did not specify their sex	26 of 164	NR	Low
Farsi [21]	2022	NA	Iran	Cross-sectional	Rome IV	PCR test/chest radiograph	Mean age (SD): 38.41 (11.51) years	Male:97 Female:136	27 of 233	NR	Low
Ghoshal [22]	2022	6 months	Bangladesh and India	Longitudinal	Rome III	RT-PCR test	Mean age (SD): SARS-CoV-2 infection group: 39.5 (15.4) years Control group: 36.8 (11.6) years	SARS-CoV-2 infection group: 204/76 (M/F) Control group: 193/71 (M/F)	20 of 280 ^a^	1 of 264 ^a^	High
Golla [23]	2022	6 months	India	Longitudinal	Rome IV	RT-PCR/CBNAAT	Mean age (SD): SARS-CoV-2 infection group: 38.02 (11.4) years Control group: 38.47 (11.7) years	SARS-CoV-2 infection group: 163/157 (M/F) Control group: 172/108 (M/F)	10 of 320	0 of 280	High
Marasco [38]	2022	6 to 12 months	Multiple countries *	Longitudinal	Rome IV	NAAT	Mean age (SD): SARS-CoV-2 infection group: 49.9 (16.1) years Control group: 50.9 (18.1) years	SARS-CoV-2 infection group: 164/105 (M/F) Control group: 364/250 (M/F)	14 of 435 ^b^	1 of 188 ^b^	High
Nazarewska [24]	2022	6 months	Poland	Longitudinal	Rome IV	NR	SARS-CoV-2 infection patients with IBS: 67.5 years old (median age) SARS-CoV-2 infection patients without IBS: 71 years old (median age)	SARS-CoV-2 infection patients with IBS: 11/4 (M/F) SARS-CoV-2 infection patients without IBS: 130/112 (M/F)	15 of 257	NR	Low
Nehme [34]	2022	12 months	Switzerland	Longitudinal	By a self-designed questionnaire	RT-PCR test	Mean age (SD): SARS-CoV-2 infection group: 44.2 (13.2) years Control group: 45.5 (14.8) years	SARS-CoV-2 infection group: 101/186 (M/F) Control group: 460/700 (M/F)	14 of 287	85 of 1160	Low
Noviello [35]	2022	5 months	Italy	Longitudinal	Rome IV	PCR test	SARS-CoV-2 infection group: 44.1 (23–60) Control group: 39.6 (22–60)	SARS-CoV-2 infection group: 98/66 (M/F) Control group: 72/111 (M/F)	43 of 164	46 of 183	High
Stepan [36]	2022	3 to 6 months	Romania	Longitudinal	Rome IV	RT-PCR test	4 to 6 years old	SARS-CoV-2 infection group: 9/14 (M/F) Control group: 2/9 (M/F)	21 of 23	6 of 11	Low
Vélez [37]	2022	6 months	USA	Longitudinal	Rome IV	NR	47.2	75/125 (M/F)	21 of 200	NR	Low

IBS, irritable bowel syndrome; SARS-CoV-2, severe acute respiratory syndrome coronavirus 2; GI, gastrointestinal; PCR, polymerase chain reaction; RT-PCR, reverse transcriptase-polymerase chain reaction; CBNAAT, cartridge- based nucleic acid amplification test; NAAT, nucleic acid amplification test; SD, standard deviation; M, male; F, female; NR, not reported; NA, not available; a, the data from the 6 months follow-up group; b, the data from the 12 months follow-up group; *, Italy, Bangladesh, Cyprus, Egypt, Israel, India, Macedonia, Malaysia, Romania, the Russian Federation, Serbia, Spain, Sweden, and Turkey.

## Data Availability

The data of this work are available from the corresponding authors.

## Supplementary Materials

The following supporting information can be downloaded at: https://www.mdpi.com/article/10.3390/jcm12051865/s1, Supplementary Materials S1: The detailed search strategies for the public databases; Supplementary Materials S2: The scores of the included study quality evaluation; Supplementary Materials S3: The results of the significance test; Supplementary Materials S4: The results of Egger’s test and Begg’s test.
