# Supplementary material for: The Prevalence of Irritable Bowel Syndrome after Severe Acute Respiratory Syndrome Coronavirus 2 Infection and Their Association: A Systematic Review and Meta-Analysis of Observational Studies
Source: J Clin Med. 2023 Feb 27;12(5):1865. doi: 10.3390/jcm12051865 (PMC10003507; doi:10.3390/jcm12051865)
Supplement: Supplementary file 1 [file jcm-12-01865-s001.zip › Supplemantary Materials S1.pdf]

Supplementary Material S1. The detailed search strategies for the public databases.

Web of Science

(COVID 19 or SARS CoV 2 or 2019 Novel Coronavirus or 2019 nCoV or Coronavirus Disease 2019 or Coronavirus Disease 19 or Severe Acute Respiratory Syndrome Coronavirus 2 or SARS Coronavirus 2) and (irritable bowel syndrome* or IBS or irritable colon*)

PubMed

(((irritable bowel syndrome) OR (IBS)) OR (irritable colon)) AND ((((((((COVID 19) OR (SARS CoV 2)) OR (2019 Novel Coronavirus)) OR (2019 nCoV)) OR (Coronavirus Disease 2019)) OR (Coronavirus Disease 19)) OR (Severe Acute Respiratory Syndrome Coronavirus 2)) OR (SARS Coronavirus 2))

Scopus

(TITLE-ABS-KEY ("COVID 19") OR TITLE-ABS-KEY ("SARS CoV 2") OR TITLE-ABS-KEY ("2019 Novel Coronavirus") OR TITLE-ABS-KEY ("2019 nCoV") OR TITLE-ABS-KEY ("Coronavirus Disease 2019") OR TITLE-ABS-KEY ("Coronavirus Disease 19") OR TITLE-ABS-KEY ("Severe Acute Respiratory Syndrome Coronavirus 2") OR TITLE-ABS-KEY ("SARS Coronavirus 2")) AND (TITLE-ABS-KEY (“irritable bowel syndrome”) OR TITLE-ABS-KEY (“IBS”) OR TITLE-ABS-KEY (“irritable colon”))

Embase

((‘irritable bowel syndrome’) OR (ibs):ti.ab,kw OR ('irritable colon')):ti.ab.kw AND ((‘sars cov2’):tiab.kw OR (‘covid19’)tiab.kw OR (‘2019 nove coronavirus’):ti,ab.kw OR (‘2019 ncov’):ti,ab.kw OR ('coronavirus disease 2019'):ti,ab.kw OR ('coronavirus disease 19'):ti,ab.kw OR ('severe acute respiratory syndrome coronavirus 2'):ti,ab,kw OR ('sars coronavirus 2')):ti,ab,kw

Cochrane Library

(COVID19 or SARS CoV2 or 2019 Novel Coronavirus or 2019 nCoV or Coronavirus Disease 2019 or Coronavirus Disease 19 or Severe Acute Respiratory Syndrome Coronavirus 2 or SARS Coronavirus 2):ti.ab.kw AND (irritable bowel svndrome or lBS or irritable colon):ti.ab.kw
