# Supplementary material for: The Prevalence of Irritable Bowel Syndrome after Severe Acute Respiratory Syndrome Coronavirus 2 Infection and Their Association: A Systematic Review and Meta-Analysis of Observational Studies
Source: J Clin Med. 2023 Feb 27;12(5):1865. doi: 10.3390/jcm12051865 (PMC10003507; doi:10.3390/jcm12051865)
Supplement: Supplementary file 1 [file jcm-12-01865-s001.zip › Supplementary Materials S2.pdf]

Supplementary Materials S2. The scores of the included study quality evaluation.

1. NOS scale

Study	Selection (0~4)	Comparability (0~2)	Exposure (0~3)	Total number of stars
Austhof [19]	3	1	2	6
Blackett [20]	4	1	2	7
Ghoshal [22]	3	1	3	7
Golla [23]	3	2	3	8
Marasco [38]	4	2	2	8
Nazarewska [24]	2	1	3	6
Nehme [34]	3	1	2	6
Noviello [35]	4	1	2	7
Stepan [36]	2	1	3	6
Vélez [37]	2	1	2	5

2. AHRQ scale

	Ebrahim [33]	Farsi [21]
1) Define the source of information (survey, record review)	Yes	Yes
2) List inclusion and exclusion criteria for exposed and unexposed subjects (cases and controls) or refer to previous publications	Yes	Yes
3) Indicate time period used for identifying patients	Yes	Yes
4) Indicate whether or not subjects were consecutive if not population-based	Yes	Yes
5) Indicate if evaluators of subjective components of study were masked to other aspects of the status of the participants	Yes	Yes
6) Describe any assessments undertaken for quality assurance purposes (e.g., test/retest of primary outcome measurements)	No	No
7) Explain any patient exclusions from analysis	Yes	Yes
8) Describe how confounding was assessed and/or controlled.	Unclear	Unclear
9) If applicable, explain how missing data were handled in the analysis	No	No
10) Summarize patient response rates and completeness of data collection	Yes	Yes
11) Clarify what follow-up, if any, was expected and the percentage of patients for which incomplete data or follow-up was obtained	Unclear	Unclear
Total items were met (total number of stars)	7	7
