# Supplementary material for: The Prevalence of Irritable Bowel Syndrome after Severe Acute Respiratory Syndrome Coronavirus 2 Infection and Their Association: A Systematic Review and Meta-Analysis of Observational Studies
Source: J Clin Med. 2023 Feb 27;12(5):1865. doi: 10.3390/jcm12051865 (PMC10003507; doi:10.3390/jcm12051865)
Supplement: Supplementary file 1 [file jcm-12-01865-s001.zip › Supplementary Materials S3.pdf]

Supplementary Material S3. The results of significance test.

1. The significance test of the IBS prevalence after SARS-CoV-2 infection [19–24,33–38].

```
Heterogeneity chi-squared = 363.17 (d.f. = 11) p = 0.000
I-squared (variation in ES attributable to heterogeneity) =  97.0%
Estimate of between-study variance Tau-squared =  0.0054

Test of ES=0 : z=   6.93 p = 0.000
```

2. The significance test of the IBS prevalence after SARS-CoV-2 infection classified by region [19–24,33–38].

```
Test(s) of heterogeneity:
                  Heterogeneity  degrees of
                    statistic     freedom      P    I-squared**   Tau-squared

North America        86.46           3      0.000     96.5%        0.0115
Asia                 15.42           2      0.000     87.0%        0.0015
Multiple Countries    0.00           0        .         .%         0.0000
Europe              236.53           3      0.000     98.7%        0.0337
Overall             363.17          11      0.000     97.0%        0.0054
** I-squared: the variation in ES attributable to heterogeneity)

Note: between group heterogeneity not calculated;
only valid with inverse variance method

Significance test(s) of ES=0

North America          z=   2.96      p = 0.003
Asia                   z=   2.93      p = 0.003
Multiple Countries     z=   3.80      p = 0.000
Europe                 z=   3.29      p = 0.001
Overall                z=   6.93      p = 0.000
```

3. The significance test of the IBS prevalence after SARS-CoV-2 infection classified by study design [19–24,33–38].

```
Test(s) of heterogeneity:
                  Heterogeneity  degrees of
                    statistic     freedom      P    I-squared**   Tau-squared

Longitudinal        337.25           9      0.000     97.3%        0.0054
Cross-sectional       1.45           1      0.228     31.1%        0.0003
Overall             363.17          11      0.000     97.0%        0.0054
** I-squared: the variation in ES attributable to heterogeneity)

Note: between group heterogeneity not calculated;
only valid with inverse variance method

Significance test(s) of ES=0

Longitudinal           z=   6.48      p = 0.000
Cross-sectional        z=   6.36      p = 0.000
Overall                z=   6.93      p = 0.000
```

4. The significance test of the association between IBS and SARS-CoV-2 infection [22,23,34–36,38].

```
Heterogeneity chi-squared =  20.94 (d.f. = 5) p = 0.001
I-squared (variation in RR attributable to heterogeneity) =  76.1%
Estimate of between-study variance Tau-squared =  0.4383

Test of RR=1 : z=   1.67 p = 0.096
```

5. The significance test of the association between IBS and SARS-CoV-2 infection classified by region [22,23,34–36,38].

```
Test(s) of heterogeneity:
               Heterogeneity  degrees of
                 statistic     freedom      P    I-squared**   Tau-squared

Asia                0.00          1       0.989     0.0%        0.0000
Multiple Countries  0.00          0         .        .%         0.0000
Europe              5.84          2       0.054    65.7%        0.1159
Overall            20.94          5       0.001    76.1%        0.4383
** I-squared: the variation in RR attributable to heterogeneity)

Note: between group heterogeneity not calculated;
only valid with inverse variance method

Significance test(s) of RR=1

Asia                 z=  3.51     p = 0.000
Multiple Countries   z=  1.75     p = 0.081
Europe               z=  0.20     p = 0.841
Overall              z=  1.67     p = 0.096
```

6. The significance test of the association between IBS and SARS-CoV-2 infection classified by study quality [22,23,34–36,38].

```
Test(s) of heterogeneity:
               Heterogeneity  degrees of
                 statistic     freedom      P    I-squared**   Tau-squared

High               18.00          3       0.000    83.3%        3.3207
Low                 6.37          1       0.012    84.3%        0.4260
Overall            20.94          5       0.001    76.1%        0.4383
** I-squared: the variation in RR attributable to heterogeneity)

Note: between group heterogeneity not calculated;
only valid with inverse variance method

Significance test(s) of RR=1

High                 z=  1.69     p = 0.092
Low                  z=  0.11     p = 0.915
Overall              z=  1.67     p = 0.096
```
