# Supplementary material for: The Prevalence of Irritable Bowel Syndrome after Severe Acute Respiratory Syndrome Coronavirus 2 Infection and Their Association: A Systematic Review and Meta-Analysis of Observational Studies
Source: J Clin Med. 2023 Feb 27;12(5):1865. doi: 10.3390/jcm12051865 (PMC10003507; doi:10.3390/jcm12051865)
Supplement: Supplementary file 1 [file jcm-12-01865-s001.zip › Supplementary Materials S4.pdf]

Supplementary Material S4. The results of Egger's test and Begg's test.

1. The prevalence of IBS after SARS-CoV-2 infection [19–24,33–38].

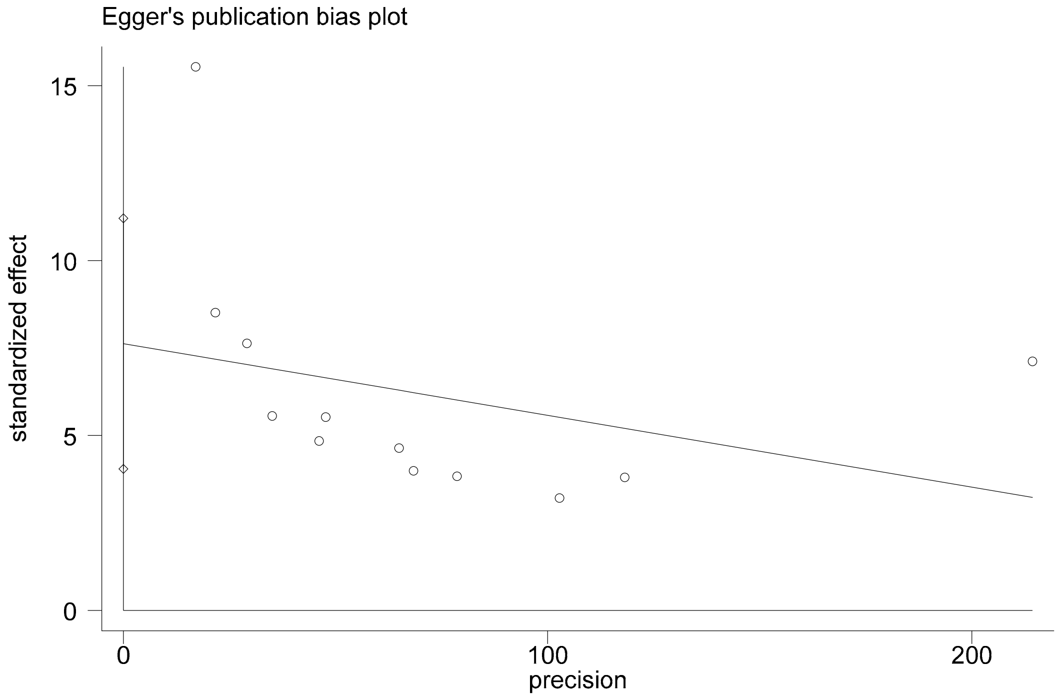

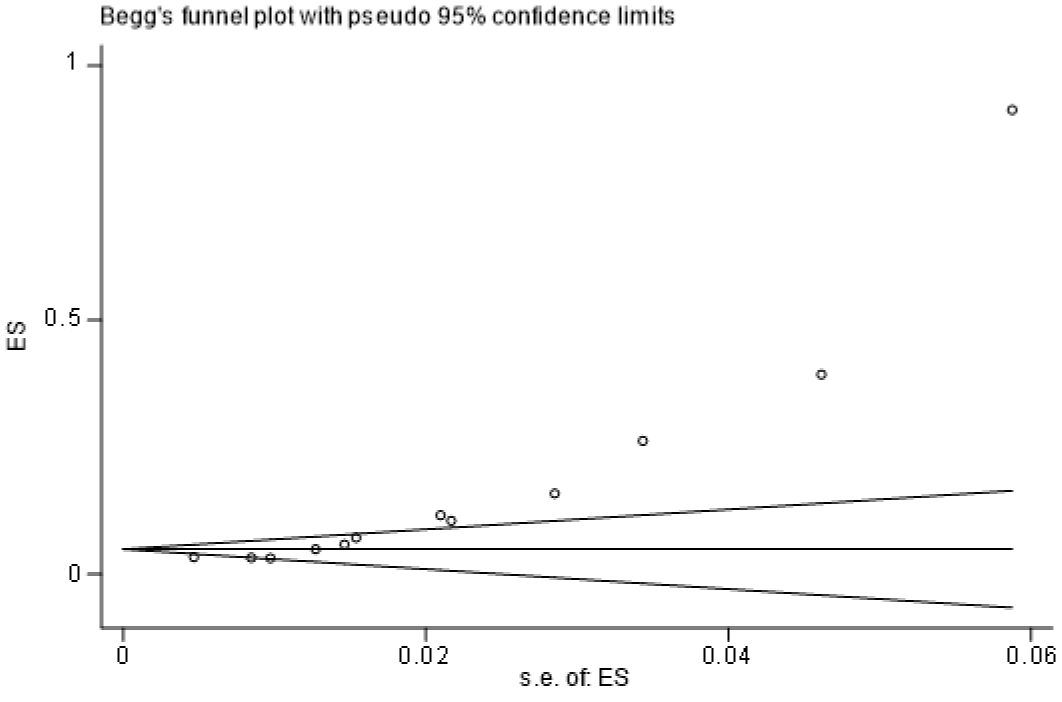

```
Tests for Publication Bias

Begg's Test

  adj. Kendall's Score (P-Q) =        64
          Std. Dev. of Score =     14.58
           Number of Studies =        12
                          z  =      4.39
                    Pr > |z| =     0.000
                          z  =      4.32 (continuity corrected)
                    Pr > |z| =     0.000 (continuity corrected)

Egger's test
```

Std_Eff	Coef.	Std. Err.	t	P>|t|	[95% Conf.	Interval]
slope	-0.0205013	0.0182814	-1.12	0.288	-0.0612347	0.0202321
bias	7.626919	1.607512	4.74	0.001	4.045159	11.20868

1. The association between IBS and SARS-CoV-2 infection [22,23,34–36,38].

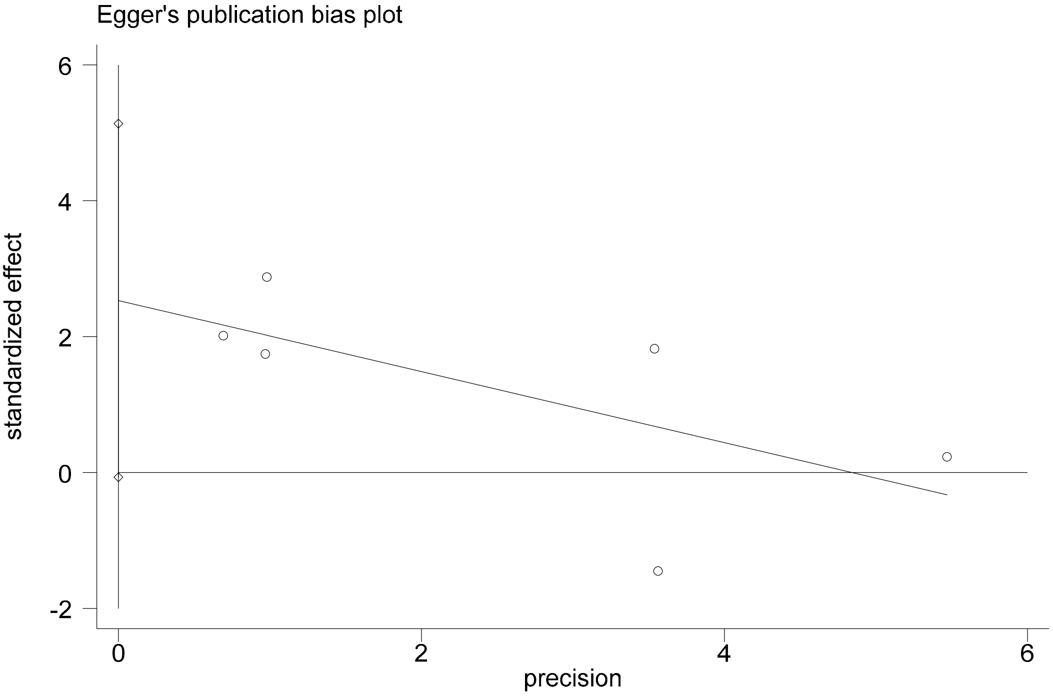

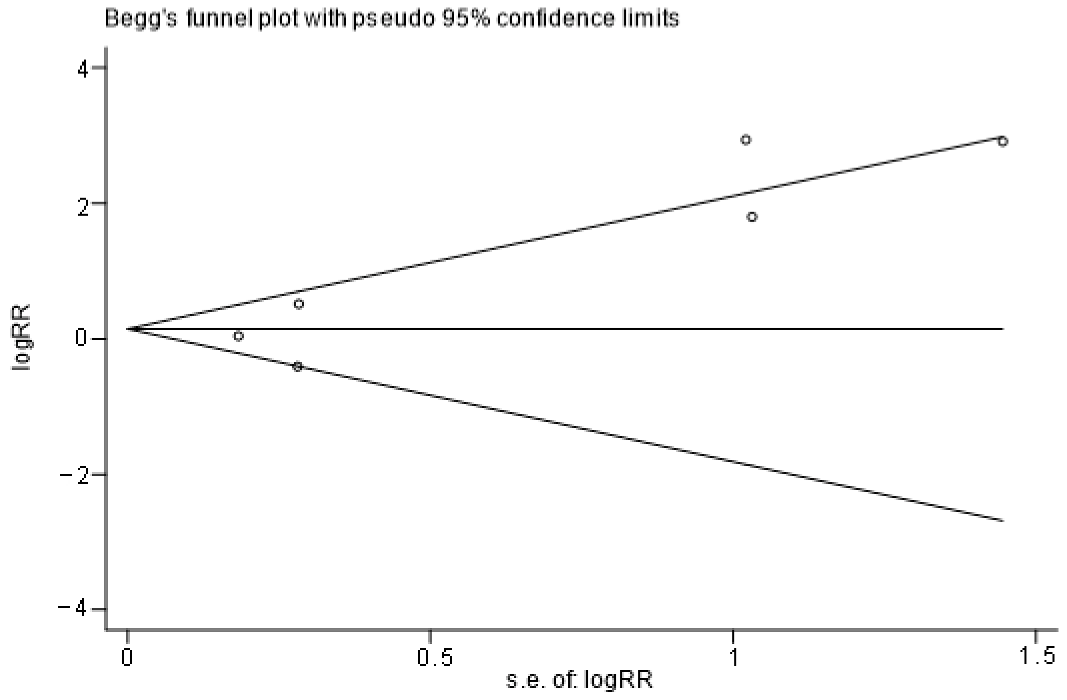


```
Tests for Publication Bias

Begg's Test

  adj. Kendall's Score (P-Q) =         9
          Std. Dev. of Score =      5.32
           Number of Studies =         6
                          z  =      1.69
                    Pr > |z| =     0.091
                          z  =      1.50 (continuity corrected)
                    Pr > |z| =     0.133 (continuity corrected)
```

Egger's test

Std_Eff	Coef.	Std. Err.	t	P>|t|	[95% Conf. Interval]	
slope	-0.5228973	0.3026561	-1.73	0.159	-1.363205	0.3174108
bias	2.532161	0.9368277	2.70	0.054	-0.0688896	5.133212
